# A Comparison of Electromyographic Inter-Limb Asymmetry During a Standard Versus a Sling Shot Assisted Bench Press Exercise

**DOI:** 10.2478/hukin-2022-0084

**Published:** 2022-09-08

**Authors:** Grzegorz Wojdala, Robert Trybulski, Marta Bichowska, Michal Krzysztofik

**Affiliations:** 1 Institute of Sport Sciences, The Jerzy Kukuczka Academy of Physical Education, Katowice, Poland; 2 Department of Medical Sciences, The Wojciech Korfanty School of Economics, Katowice, Poland; 3 Provita Zory Medical Center, Zory, Poland; 4 Faculty of Physical Education, Gdansk University of Physical Education and Sport, Gdansk, Poland

**Keywords:** resistance exercise, EMG, training equipment, limb symmetry index, internal movement structure

## Abstract

The objective of this study was to compare peak surface electromyography (sEMG) activity of selected muscles along with inter-limb asymmetries between a control (CONT) and a Sling shot assisted (SS) bench press exercise. Ten resistance-trained males with at least three-year experience in resistance training (22.2 ± 1.9 years, 88.7 ± 11.2 kg, 179.5 ± 4.1 cm, bench press one-repetition maximum (1RM) = 127.3 ± 25.9 kg) performed the flat bench press exercise under two conditions at selected loads (85% and 100% of 1RM assessed without the SS). Peak sEMG amplitude of triceps brachii, pectoralis major, and anterior deltoid was recorded for the dominant and the non-dominant side of the body during each attempt. The comparison between the dominant and the non-dominant side was carried out using the limb symmetry index (LSI(%) = (2*(XR - XL)/(XR + XL))*100%) where XR = values of the right side and XL = values of the left side. There was a main effect of condition (p = 0.004; η^2^ = 0.64) and the load (p = 0.004; η^2^ = 0.63) for the triceps brachii LSI in parallel with a main effect of condition (p = 0.003; η^2^ = 0.42) for the anterior deltoid LSI. Post hoc analysis for the main effect of condition showed significant differences in the LSI between the CONT and SS conditions for the triceps brachii (p = 0.003; 1.10% vs. -8.78%) as well as for the anterior deltoid muscles (p = 0.03; 12.91% vs. 9.23%). The results indicate that the assistance of the Sling shot significantly affects the sEMG activity pattern on both the dominant and non-dominant sides of the body while influencing inter-limb asymmetries.

## Introduction

Inter-limb asymmetries have been the topic of interest in recent years, mainly considering gait analysis and lower limb electromyography comparison (Abdul Halim et al., 2019; Bishop et al., 2018). The determination of inter-limb asymmetry frequently demands the assessment of symmetry indexes for proper analysis and correct inference. Such equations enable to evaluate the extent and the direction of the asymmetry suggested to be highly task-specific, mainly described as a percent value (Bishop et al., 2018; Carpes et al., 2010). Referring to empirical data, a significant asymmetry of muscle activity and strength causes greater loading on passive structures and results in limited recovery with a greater risk of injury (Croisier et al., 2008), which can cause a decrease in physical performance (Bishop et al., 2018). Nevertheless, the degree of imbalances will vary depending on the performed activity and the choice of unilateral or bilateral exercise (Kuki et al., 2019). On the contrary, asymmetry is a natural feature, associated with anatomical and neurological factors, therefore it can be considered functional (Raya-González et al., 2021). The importance of muscle asymmetry, as well as imbalances in the kinematic, kinetic and muscle excitation of upper limbs in relation to overall physical or sports performance have not been sufficiently researched. Concerning the asymmetry of muscle excitation patterns during bilateral upper body resistance exercises such as the barbell bench press, the majority of researchers have based their conclusions on surface electromyography (sEMG) analysis from the dominant side of the body (Stastny et al., 2017). Regarding most recent bench press research in competitive athletes as well as in recreationally trained subjects, authors often indicate the necessity of measuring sEMG activity on both sides of the body due to significant differences of peak sEMG amplitudes indicating higher values on the dominant side (Gołaś et al., 2018; Jarosz et al., 2020; Krzysztofik et al., 2021). The difference in muscle excitation between the dominant and non-dominant sides is manifested not only in the sEMG activity, but also in movement velocity, strength, consistency of movement and delayed fatigue (Bravi et al., 2017). Furthermore with an increase in external loads, an increase in sEMG amplitude is partially related with stabilization requirements, which may eventually lead to an increase in inter-arm asymmetry (Gołaś et al., 2018). The occurrence of a certain amount of sEMG activity asymmetry is also attributed to previous injuries, muscle imbalances or limb dominance; therefore, unilateral analysis based on one side of the body may result in inconsistencies and misinterpretations (Gołaś et al., 2018; Krzysztofik et al., 2021).

Recently, innovative approaches to developing or modifying exercises focused on strength and power output have emerged. An increasingly common phenomenon in advanced training is the use of elastic resistance and assisted equipment (Bellar et al., 2011; Dugdale et al., 2019; Wilk et al., 2020b). The application of elastic resistance consists in using various flexible bands to challenge a movement pattern and adjust the force capability of the muscles across the range of motion, whereas elastic assistance training uses a supportive or an overspeed approach allowing to perform supramaximal effort (Dugdale et al., 2019; Wilson and Kritz, 2014). While there is extensive literature on elastic resistance (Bellar et al., 2011; McMaster et al., 2009; Swinton et al., 2014), far less attention has been given to the use of elastic assistance. The implementation of this method for the upper body can be done using a supportive device called the Sling shot. The Sling shot is made of extensible fabric with two connected sleeves, which makes it elastic and resilient while providing a braking effect on movement during the eccentric phase. Generally the Sling shot is a passive element, but during movement (especially in the eccentric contraction), the strain of the material of which the Sling shot is made ensures additional elastic energy which assists the athlete during the eccentric phase of movement providing a “rebound” effect during the concentric phase of the lift, while increasing the lifted load and power output (Wilk et al., 2020b, 2020c; Wojdala et al., 2020). The utilization of the Sling shot increases one-repetition maximum (1RM) test results (Dugdale et al., 2019; Ye et al., 2014), increases the maximal number of performed repetitions (Niblock and Steele, 2017; Pedrosa et al., 2020), bar velocity and power output (Dugdale et al., 2019; Ye et al., 2014) as well as changes in sEMG activity of the prime movers (Dugdale et al., 2019; Wojdala et al., 2020; Ye et al., 2014). It was evidenced that the Sling shot used during the bench press caused a decrease in sEMG of the prime movers, however, the degree of these changes depends upon the external load and the muscles examined (Wojdala et al., 2020).

Previous research on the impact of the Sling shot on sEMG activity concerned only the dominant side of the body (Dugdale et al., 2019; Wojdala et al., 2020; Ye et al., 2014). This seems a major limitation of those studies due to significant differences in sEMG amplitude between the dominant and non-dominant limbs (Gołaś et al., 2018; Jarosz et al., 2020). Therefore, a comprehensive study is needed on the analysis of sEMG activity changes in the dominant and non-dominant sides and muscle asymmetry occurring during dynamic exercises. Thus, the purpose of this study was to evaluate the acute impact of the Sling shot on the inter-limb asymmetry determined by the sEMG activity during the bench press exercise at submaximal and maximal external loads. Since the prime movers involved during the bench press are the pectoralis major, triceps brachii and anterior deltoid (Stastny et al., 2017), these muscle groups were selected for evaluation. It was hypothesized that the application of the Sling shot, through its structure and properties, would decrease inter-limb asymmetry. If so, it would create opportunities to use the Sling shot as a rehabilitation tool to maximize athletic performance and reduce the risk of injury. Moreover, considering that previous studies have shown that the Sling shot causes a decrease in sEMG activity of the dominant side of the body (Dugdale et al., 2019; Wojdala et al., 2020; Ye et al., 2014), it was expected that the application of the Sling shot would reduce the sEMG activity of both the dominant and non-dominant limbs. The sample and the design, together with the research data, have been reported in an earlier publication (Wojdala et al., 2020). However, the present study extends the previous experiment by examining the unused data of the non-dominant side of the body along with calculating the limb symmetry index (LSI).

## Methods

### Experimental Approach to the Problem

The study was carried out according to a randomized crossover design, where each participant attended two experimental sessions: with the Sling shot (SS) and without it, as a control condition (CONT) separated by a one-week interval. During each of the experimental bench press sessions the participant performed a single repetition with a load of 85% and 100% 1RM evaluated without the Sling shot. The anterior deltoid, triceps brachii and pectoralis major peak sEMG amplitudes were recorded during both sessions. The comparison between the right and the left side was carried out using the limb symmetry index (LSI(%) = (2*(X_R_ - X_L_)/(X_R_ + X_L_))*100%) where X_R_ = values of the right side and X_L_ = values of the left side (Aedo-Muñoz et al., 2019; Bishop et al., 2018). A positive LSI value indicated superiority of the right side, while a negative value showed superiority of the left side, whereas a score of 0 would indicate perfect symmetry between the limbs (Carpes et al., 2010).

One week before experimental sessions, participants completed a familiarization session including the 1RM bench press test protocol. Participants were required to withdraw from resistance training 72 h prior to each experimental session. Furthermore, participants were asked to maintain their dietary habits and sleep hygiene, refrain from consuming alcohol and taking ergogenic aids or medications for 24 h prior to, and throughout the experimental sessions.

### Participants

Ten resistance-trained male subjects participated in the study. Their age, 1RM in the bench press, body height and body mass equaled 22.2 ± 1.9 years, 127.3 ± 25.9 kg, 88.7 ± 11.2 kg and 179.5 ± 4.1 cm, respectively. The minimum resistance training experience required to participate in the study equaled 3 years, with an average of 6.0 ± 2.5 years. It should be emphasized that right-handedness with the right upper limb domination was found in all study participants. Participants were informed about the benefits and potential risks of the study prior to commencement of the experiment and gave their written consent to participate. Participants did not report any injuries or musculoskeletal disorders at the time of the study and were free to withdraw from the study at any moment. All measurements were conducted in the Strength and Power Laboratory of the Academy of Physical Education in Katowice. The research received the approval of the Bioethics Committee for Scientific Research, at the Academy of Physical Education in Katowice, Poland (3/2021) and was performed in accordance with the ethical standards of the Declaration of Helsinki, 2013.

### Design and Procedures

The 1RM test was performed as previously described (Seo et al., 2012; Wojdala et al., 2020), yet without the Sling shot assistance. Testing was scheduled for the same time of the day for all experimental sessions to minimize the effects of the circadian rhythm. Testing started with dynamic mobility exercises for the upper body preceded by a general warm-up on the cycle ergometer for 5 min (heart rate of around 130 beats per minute). Afterwards, the specific part of the warm-up was carried out which consisted of 15, 10, and 5 bench press repetitions using 20%, 40%, and 60% of the estimated 1RM, respectively (Krzysztofik et al., 2020). The bench press grip width used for all experimental sessions was set at 150% of each participant’s bi-acromial distance (Wilk et al., 2019). The 1RM test consisted of four to six attempts, starting with a load of 70% estimated 1RM. In each subsequent attempt, participants performed a single repetition using a 2/0/V/0 tempo of movement. These values refer to a 2 s negative work of lowering the barbell, a lack of a pause in the transition phase, and a volitional movement tempo while lifting the barbell during positive work (Wilk et al., 2020a). The load was increased by 2.5-10 kg for each consecutive attempt and the process was repeated until failure with a 5 min rest interval before each repetition. Each repetition was executed while maintaining the hips on the bench and without bouncing the bar off of the chest.

**Figure 1:**
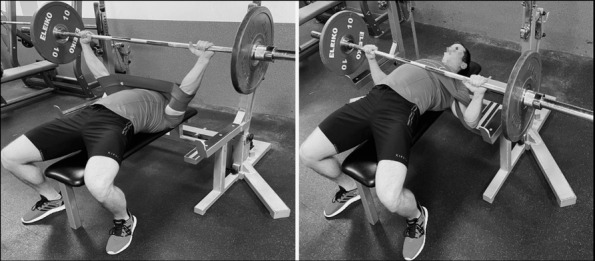
Position of the Sling shot during the sample repetition of the barbell bench press; the Sling shot sleeves were located in the middle of the elbows.

The familiarization session was used to select the appropriate size of the Sling shot and to get participants familiarized with the Sling shot assisted bench press exercise paying attention to the technique of the movement execution. The Sling shot size was adopted based on bodyweight and manufacturer's guidelines (extra-large, large and medium size, each providing the same tension). In order to assess the technical proficiency, the correct movement technique was demonstrated by a resistance training coach together with the proper Sling shot placement (Figure 1). Following the general and specific warm-up, participants were allowed to practice the assisted bench press repeatedly until they felt comfortable performing the exercise (Wojdala et al., 2020; Ye et al., 2014). Afterwards, participants performed four sets of a single bench press repetition with the Sling shot using 80% 1RM.

**Figure 2:**
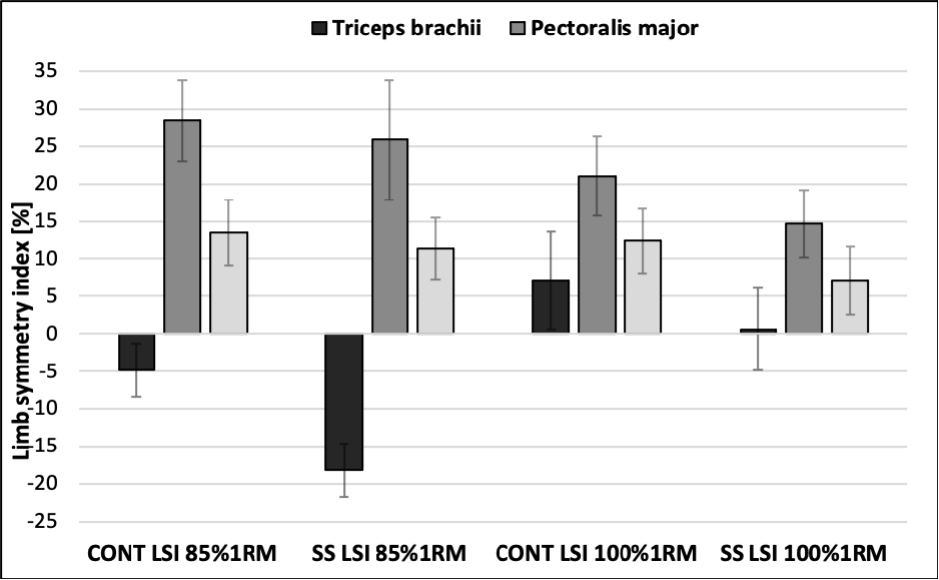
Comparison of the limb symmetry index of muscles recorded under different conditions and with different external loads.

### Experimental Session

In a randomized crossover design each participant attended two experimental bench press sessions according to the SS and CONT protocols. During the experimental sessions participants performed a single repetition at a load of 85% and 100% 1RM assessed without the Sling shot, with five-minute rest intervals between particular trials. Grip width, rack heights, tempo of movement and the warm-up protocol were the same as in the familiarization session.

### Electromyography

Peak sEMG amplitude of pectoralis major, triceps brachii and anterior deltoid muscles was collected and analyzed bilaterally with an eight-channel Noraxon TeleMyo 2400 system (Noraxon USA Inc., Scottsdale, AZ, USA; 1500 Hz). The gel-coated self-adhesive electrodes (Dri-Stick Silver circular sEMG Electrodes AE-131, NeuroDyne Medical, USA), with a 11 mm contact diameter and a 2 cm center-to-center distance, were located along the assumed direction of the underlying muscle fibers with reference to the SENIAM recommendations (Konrad, 2006). The skin at the measurement spot was previously abraded, shaved and washed with alcohol. The grounding electrode was placed on the connection with the anterior deltoid muscle. In order to ensure repeatability of the mounting position, landmarks were used to place the electrodes during subsequent trials. The sEMG signals were collected with a sampling frequency of 1000 Hz and bandpass filtered (8-450 Hz), then subjected to a moving 100 ms root mean square (RMS) window and respectively normalized to the peak sEMG amplitude. The maximum voluntary isometric contractions (MVICs) were recorded for both sides of the body separately before and after each experimental session. Testing positions were chosen on the basis of the SENIAM procedure (Konrad, 2006), and standardized protocols (Stastny et al., 2017). The pectoralis major MVICs were recorded at an isometric Smith machine bench press immobilized by the supramaximal load with the arm abducted and the elbow flexed to 90°, triceps brachii MVICs at the seated triceps extension at 90° elbow flexion and anterior deltoid MVICs using a seated shoulder abduction with 90% arm flexion (Konrad, 2006). Participants gradually increased the force of the muscle contraction for two seconds and then generated maximum tension for three seconds. The MVIC of each examined muscle was selected to normalize sEMG results. Furthermore, the highest peak sEMG amplitude of the entire bench press repetition was used to estimate a percentage of MVIC (%MVIC).

### Statistical Analysis

All statistical analyses were performed using Statistica 9.1. Results are presented as means with standard deviations. The Shapiro-Wilk test was used in order to verify the normality, homogeneity, and sphericity of the sample data variances. Differences in %MVIC between the CONT and SS conditions were examined using repeated measures three-way ANOVA (2 conditions [CONT vs. SS] × 2 loads [85% 1RM vs. 100% 1RM] × 2 side [right vs. left]). Furthermore, a two-way ANOVA (2 conditions × 2 loads) was used to compare LSI values. An independent analysis was performed for each muscle. Effect sizes for main effects and interactions were determined by partial eta squared (η^2^). Partial eta squared values were classified as small (0.01 to 0.059), moderate (0.06 to 0.137) and large (> 0.137). Post hoc comparisons using the Tukey’s test were conducted to locate the differences between mean values when a main effect or an interaction was found. For pairwise comparisons, effect sizes were determined by Cohen’s *d* which was characterized as large (*d* > 0.8), moderate (*d* between 0.8 and 0.5), small (*d* between 0.49 and 0.20) and trivial (*d* < 0.2) (Cohen, 1988). Percent changes with 95% confidence intervals (95CI) were also calculated. Statistical significance was set at *p* < 0.05.

## Results

### %MVIC of triceps brachii

The three-way repeated-measures ANOVA showed a statistically significant interaction for %MVIC condition × side; (*p* = 0.003; η^2^ = 0.62) and for load × side (*p* = 0.004; η^2^ = 0.63). The post hoc for interaction condition × side showed significantly higher %MVIC for the CONT right side and the CONT left side when compared to the SS right side and the SS left side (*p* < 0.001 for all). We also registered significantly higher %MVIC for the SS left side when compared to the SS right side (*p* = 0.009). The post hoc tests for interaction of load × side showed significantly lower %MVIC for the load of 85% 1RM right side when compared to the load of 85% 1RM left side (*p* = 0.02), 100% 1RM right side (*p* < 0.001) and 100% 1RM left side (*p* < 0.001). We also observed significantly lower %MVIC results for the load of 85% 1RM left side (*p* = 0.02), compared to 100% 1RM right side (*p* < 0.001) and 100% 1RM left side (*p* < 0.001). There were no differences in %MVIC between the load of 100% 1RM right side and 100% 1RM left side (*p* = 0.31).

We also registered a significant main effect for condition (*p* < 0.001; η^2^ = 0.96) and for load (*p* < 0.001; η^2^ = 0.96). Post hoc tests for the main effect of condition showed significantly higher %MVIC for the CONT when compared to the SS condition (*p* < 0.001). Post hoc tests for the main effect of load showed significantly higher %MVIC for the load of 100% 1RM when compared to 85% 1RM (*p* < 0.001).

### %MVIC of the pectoralis major

The three-way repeated-measures ANOVA showed a statistically significant interaction for %MVIC condition × side (*p* = 0.02; η^2^ = 0.46). Post hoc test results for the interaction of condition × side showed significantly higher %MVIC for the CONT right side, when compared to the CONT left side, the SS right side and the SS left side (*p* < 0.001 for all), significantly higher %MVIC for the CONT left side compared to the SS right side and the SS left side (*p* < 0.001 for all), significant higher %MVIC for the SS right side when compared to the SS left side (*p* < 0.001).

There was also a significant main effect for condition (*p* < 0.001; η^2^ = 0.85), load (*p* < 0.001; η^2^ = 0.96) and side (*p* < 0.001; η^2^ = 0.94). Post hoc test results for the main effect of condition showed significantly higher %MVIC for the CONT when compared to the SS condition (*p* < 0.001). Post hoc results for the main effect of load showed significantly higher %MVIC for the load of 100% 1RM when compared to 85% 1RM (*p* < 0.001). Post hoc tests for the main effect of side showed significantly higher %MVIC for the right side in comparison to the left side (*p* < 0.001).

### %MVIC of anterior deltoid

The three-way repeated-measures ANOVA showed a statistically significant interaction for %MVIC condition × load; (*p* = 0.02; η^2^ = 0.48), and for condition × side (*p* = 0.008; η^2^ = 0.55). Post hoc tests for the interaction of condition × load showed significant differences between CONT 85% 1RM, CONT 100% 1RM, SS 85% 1RM and SS 100% 1RM (*p* < 0.001 for all). Post hoc tests for the interaction of condition × side showed significantly higher %MVIC for the CONT right side when compared to the CONT left side, the SS right side and the SS left side (*p* < 0.001 for all). Also, we observed significantly higher %MVIC for the CONT left side when compared to the SS left side (*p* < 0.001), and significantly higher %MVIC for the SS right side when compared to the SS left side (*p* < 0.001).

There was also a significant main effect for condition (*p* < 0.001; η^2^ = 0.97) of load (*p* < 0.001; η2 = 0.81) and side (*p* < 0.001; η^2^ = 0.71). Post hoc tests for the main effect of condition revealed significantly higher %MVIC for the CONT when compared to the SS condition (*p* < 0.001). Post hoc tests for the main effect of load showed significantly higher %MVIC for the load of 100% 1RM when compared to 85% 1RM (*p* < 0.001). The post hoc tests for the main effect of side showed significantly higher %MVIC for the right when compared to the left side (*p* = 0.001).

### LSI triceps brachii

The two-way repeated-measures ANOVA showed a statistically significant main effect for condition (*p* = 0.004; η^2^ = 0.64) and for load (*p* = 0.004; η^2^ = 0.63). Post hoc tests for the main effect of condition showed significant differences in the LSI between the CONT and SS conditions (*p* = 0.003; 1.10% vs. -8.78% respectively). Post hoc tests for the main effect of load showed significant differences in the LSI between the 85% 1RM and 100% 1RM load (*p* = 0.004; -11.53% vs. 3.85% respectively).

### LSI pectoralis major

The two-way repeated-measures ANOVA did not show a statistically significant interaction (*p* = 0.52; η^2^ = 0.04) and did not show a significant main effect for condition (*p* = 0.15; η^2^ = 0.21) and for load (*p* = 0.11; η^2^ = 0.25).

### LSI anterior deltoid

The two-way repeated-measures ANOVA showed a statistically significant main effect for condition (*p* = 0.003; η^2^ = 0.42). Post hoc tests for the main effect of condition showed significant differences in the LSI between the CONT and SS conditions (*p* = 0.03; 12.91% vs. 9.23, respectively).

## Discussion

The main finding of the present research indicates that inter-limb asymmetries, determined by the LSI formula, differed significantly between the CONT and SS conditions. The Sling shot assistance resulted in decreased LSI values for the anterior deltoid and triceps brachii muscles. Furthermore, the LSI of the triceps brachii muscle increased with progressing external loads, while the asymmetry of the pectoralis major was not affected by the conditions or the load used. Furthermore, there was a significant interaction between the conditions, the load and the measured side and peak sEMG amplitude within particular muscles. The excitation of all measured muscles on both sides was dependent on the applied external load and the condition, increasing up to the load of 100% 1RM while decreasing under the SS condition. The obtained results showed a significantly higher %MVIC of the anterior deltoid and pectoralis major muscles on the dominant side at both loads regardless of the condition. On the contrary, comparing the triceps brachii muscle sEMG activity at 85% 1RM, the non-dominant side demonstrated a higher %MVIC, both during the CONT as well as the SS protocol. Therefore, the results confirm the hypothesis that the Sling shot has a significant impact on limb asymmetries, yet the influence differs depending on the external load and the muscle examined.

Most previous studies that evaluated changes in sEMG activity during the bench press assessed only the dominant side of the body/limb (Stastny et al., 2017). Nevertheless, despite the lack of precise guidelines regarding bilateral measurements (Besomi et al., 2020), researchers have recently attempted an evaluation of body asymmetry and laterality. Several authors have mentioned the need to measure sEMG activity on both the right and the left side of the body as the sEMG amplitudes differed significantly, pointing out increased values on the dominant side (Gołaś et al., 2018; Jarosz et al., 2020; Krzysztofik et al., 2021). This is in line with the results of this study which showed significant differences in sEMG activity between the dominant and the non-dominant side of the body. Such differences were observed for both conditions. Greater muscle tension on the dominant side, which was present in the pectoralis major and anterior deltoid, confirms the predominance of one limb over the other (Gołaś et al., 2018; Krzysztofik et al., 2021). Possible explanations are related to persistent preferential use of the dominant limb leading to morphological and physiological adaptations in muscle function and composition, enlarged excitability of the dominant motor cortex or central nervous system optimization (Bravi et al., 2017; Williams et al., 2002). Furthermore, motor lateralization reflects the proficiency of each arm for complementary functions, with the dominant arm relying on dynamic properties of movement and the non-dominant arm on optimizing positional stability (Mutha et al., 2013). These dependencies apply to the entire limb, but not necessarily to particular muscles involved in specific movements (Gołaś et al., 2018), as confirmed in our study by greater triceps brachii sEMG activity on the non-dominant side with submaximal loads, which may have been compensated by substantially increased excitation of the pectoralis major on the dominant side. The LSI formula indicated that the SS condition significantly altered the inter-limb sEMG amplitude ratio for both the triceps brachii and anterior deltoid muscles relative to the CONT condition. The results also indicate an increased involvement of the non-dominant limb in the Sling shot assisted exercise, which was confirmed by lower LSI values with a particularly high decrease of the triceps brachii LSI (Table 2). However, it should be taken into account that of the prime movers, the triceps brachii sEMG amplitude is the most susceptible to change during various conditions of the bench press exercise (Krzysztofik et al., 2020; Stastny et al., 2017). The contrast in sEMG activities of particular muscles on both sides of the body likely reflects different levels of muscular strength, acquired movement patterns through long term training or past injuries (Gołaś et al., 2018). Thereby, it seems advisable to analyze the sEMG activity of chosen muscles during resistance exercises on both sides of the body.

**Table 1:** Peak sEMG amplitude of muscles recorded for both sides of the body under different conditions and with different external loads.

Muscle group	%MVIC RIGHT SIDE	%MVIC LEFT SIDE	ES	%MVIC RIGHT SIDE	%MVIC LEFT SIDE	ES
	(95% CI)	(95% CI)		(95% CI)	(95% CI)	
	85% 1 RM CONT			85 % 1RM SS		
Anterior	114.6 ± 8.1	100.1 ± 6.1	2.02	101.1 ± 7.6	90.1 ± 4.5	1.76
deltoid	(108.8 to 120.4)	(95.7 to 104.5)		(95.7 to 106.5)	(86.9 to 93.3)	
Pectoralis	66.4 ± 5.6	49.9 ± 4.9	3.14	56.6 ± 4.5	43.6 ± 3.3	3.29
major	(62.4 to 70.4)	(46.4 to 53.4)		(53.4 to 59.8)	(41.2 to 46.0)	
Triceps brachii	79.8 ± 6.1	83.7 ± 4.6	0.72	59.4 ± 5.6	71.1 ± 3.1	2.59
	(75.4 to 84.2)	(80.4 to 87)		(55.4 to 63.4)	(68.9 to 73.3)	
	100% 1RM CONT			100 % 1RM SS		
Anterior	122.5 ± 6.0	108.3 ± 7.2	2.14	104.2 ± 4.0	97.2 ± 6.8	1.25
deltoid	(118.2 to 126.8)	(103.2 to 113.4)		(101.3 to 107.1)	(92.3 to 102.1)	
Pectoralis	86.6 ± 6.3	70.5 ± 9.5	2.00	73.0 ± 4.8	63.0 ± 4.2	2.22
major	(82.1 to 91.1)	(63.7 to 77.3)		(69.6 to 76.4)	(60.0 to 66.0)	
Triceps brachii	103.5 ± 5.5	96.4 ± 5.2	1.33	84.4 ± 8.6	83.5 ± 2.7	0.14
	(99.6 to 107.4)	(92.7 to 100.1)		(78.3 to 90.5)	(81.6 to 85.4)	

Data are presented as mean ± standard deviation and 95% confidence interval (95% CI); CONT = control condition; SS = Sling shot condition; MVIC = maximum voluntary isometric contractions; ES = effect size.

**Table 2:** The limb symmetry index of muscles recorded under different conditions and with different external loads.

Muscle group	LSI CONT (95% CI)	LSI SS (95% CI)	ES	LSI CONT (95% CI)	LSI SS (95% CI)	ES
	85% 1RM			100% 1RM		
Anterior	13.4 ± 8.7	11.4 ± 8.5	0.24	12.4 ± 8.1	7.1 ± 9.1	0.61
deltoid	(7.2 to 19.7)	(5.3 to 17.5)		(6.6 to 18.2)	(0.6 to 13.6)	
Pectoralis	28.4 ± 10.8	25.9 ± 10.6	0.24	21.0 ± 16.0	14.7 ± 9.0	0.49
major	(20.7 to 36.1)	(18.3 to 33.5)		(9.6 to 32.4)	(8.2 to 21.2)	
Triceps brachii	-4.9 ± 7.2	-18.2 ± 13.0	1.26	7.1 ± 7.1	0.6 ± 10.9	0.71
	(-10.0 to 0.2)	(-27.5 to -8.8)		(2.0 to 12.2)	(-7.2 to 8.4)	

Data are presented as mean ± standard deviation and 95% confidence interval (95% CI); LSI =limb symmetry index; CONT = control condition; SS = Sling shot condition; ES = effect size.

The results of the current study demonstrated significant differences in the bench press prime movers’ peak sEMG amplitude between the sides of the body and various loads, concurrently evaluating separate lifting conditions considering the Sling shot assistance. The %MVIC values of all prime movers obtained under the SS condition were significantly lower than those in the CONT protocol, which is consistent with previous studies (Dugdale et al., 2019; Wojdala et al., 2020; Ye et al., 2014). However, this is the first study where this phenomenon has also been confirmed for peak sEMG amplitude in the non-dominant limb. Depending on the external load, side and muscle analyzed, the decrease in peak sEMG amplitude using the Sling shot ranged from 6.3 to 20.4 %MVIC (Table 1). This reduction occurs as a result of the elastic assistance enhancement of the Sling shot on both sides of the body mainly by generating greater initial bar velocity and decreasing the time under tension of each repetition (Pedrosa et al., 2020) as an essential factor of muscle excitation (Wilk et al., 2020d). Although the decline in sEMG amplitude was recorded for all measured muscles, it should be emphasized that the greatest decrease caused by the Sling shot assistance occurred in the triceps brachii muscle (12.6 to 20.4 %MVIC; Table 1), which possibly results from the placement of the sleeves. Dugdale et al. (2019) suggest that the Sling shot directly influences the elbow position causing a change in bench press mechanics and the occurrence of the sticking point, changing the triceps brachii sEMG activity. This is probably correlated with the largest stretch of the fabric and mechanical assistance at the start of the positive work during the bench press repetition where the involvement of the triceps brachii muscle is fundamental (Dugdale et al., 2019; Van Den Tillaar and Ettema, 2009, 2010; Wojdala et al., 2020). The triceps brachii LSI evaluated in the current study was susceptible to an increase in the external load as indicated by significantly lower asymmetry along with greater involvement of the dominant limb at the maximum load, whereas the difference between the loads was greater for the SS compared to the CONT condition (Table 2). This is partly consistent with the study by Gołaś et al. (2018) which showed a reduced inter-limb difference in total sEMG amplitude of the bench press at a load of 90% 1RM compared to 70% 1RM. Those authors explained this relationship by higher activation of the central nervous system and optimized muscle coordination to perform a more demanding motor task according to the Henneman's size principle (Henneman et al., 1965; Strońska et al., 2018). The use of the Sling shot as a training tool, through reducing the muscle excitation of prime movers, allows to train through a larger volume while generating less stress on the elbow and shoulder joints what may convert into greater gains in muscle strength (Niblock and Steele, 2017; Ye et al., 2014). Furthermore, considering the greatest decrease in peak sEMG amplitude of the triceps brachii during the bench press with the Sling shot, additional complementary exercises of this muscle group should be implemented in order to maintain optimal strength and muscular hypertrophy (Peterson et al., 2011; Wojdala et al., 2020).

Among all the prime movers, only the pectoralis major LSI showed no significant differences between conditions or loads together with a relatively lower %MVIC compared to the other muscles examined (Table 2). It may be attributed to a change in the bench press movement pattern at loads close to maximal when the pectoralis major changes from a prime mover to a supportive prime mover limiting its contribution to movement (Król and Gołaś, 2017). Moreover, it is also suggested that the Sling shot does not affect the pectoralis major muscle inter-limb symmetry considering the Sling shot position directly on the chest and a negligible moment of force. Furthermore, it is noticeable in the present study that the pectoralis major demonstrated the highest baseline LSI values under both the CONT and SS conditions. Such results are in accordance with the study of Aedo-Muñoz et al. (2019) who conducted research on Paralympic weightlifters including LSI assessment in relation to the bench press. Results of that study confirmed the possibility of inter-limb asymmetries exceeding 20% in a group of resistance trained subjects, nonetheless, it should be noted that individual values can vary significantly. However, there are no conclusive recommendations as to the optimal ranges of the LSI for preventing injury or optimizing athletic performance considering the bench press exercise, thus research in this area should be continued.

There are some study limitations that need to be addressed. The research findings proved significant changes of the prime movers sEMG activity, though the stabilizer and antagonistic muscles were not included in the analysis of the internal structure of the movement. Furthermore, the kinematics of both bench press conditions along with the external structure of the movement (i.e., forces and movement torques) were not examined in this study. Relative instead of absolute loads were used for the evaluation, with no 1RM measurements with the Sling shot assistance. Future research should address the influence of the Sling shot on stabilizer muscles in the bench press in both men and women, as well as the impact of the inter-limb asymmetries together with strength, power and hypertrophy adaptations.

## Conclusions

The results of the present study indicate that the Sling shot assistance significantly affects the sEMG activity pattern on both the dominant and non-dominant sides of the body while influencing the inter-limb asymmetries. The LSI of the prime movers considered during the bench press exercise implies that the Sling shot assistance increases the relative involvement of the non-dominant limb while decreasing the sEMG muscle activity of both limbs. Furthermore, the load increase was associated with greater symmetry of movement, mainly due to the shift of excitation of the triceps brachii muscle. Significant asymmetries between the limbs justify the recording of sEMG activity on both sides of the body, which should be the basis for modern research using sEMG.
